# Pedagogical Applications, Prospects, and Challenges of Blended Learning in Chinese Higher Education: A Systematic Review

**DOI:** 10.3389/fpsyg.2021.772322

**Published:** 2022-01-25

**Authors:** Muhammad Azeem Ashraf, Shorif Mollah, Shahnaz Perveen, Nadia Shabnam, Lizoon Nahar

**Affiliations:** ^1^Research Institute of Educational Science, Hunan University, Changsha, China; ^2^Smart Learning Institute, Beijing Normal University, Beijing, China; ^3^Department of Education, The Government Sadiq College Women University Bahawalpur, Bahawalpur, Pakistan; ^4^Department of Health Profession Education, National University of Medical Sciences, Rawalpindi, Pakistan; ^5^College of Education and Communications, Indiana University of Pennsylvania, Indiana, PA, United States

**Keywords:** hybrid learning, blended learning, B-learning, framework, Moodle, learning management system (LMS), pedagogy

## Abstract

In recent years, blended learning (BL) has grown to occupy an important space in Chinese educational practice. Policymakers have developed many application strategies and platforms and are continuing to develop BL for future use. In order to apply BL in practice, key stakeholders have been using different learning management systems (LMSs), digital platforms, games, hybrid courses, and various forms of social media to create a framework for BL. This study asserts that many visible opportunities have emerged in Chinese higher education through the applications of BL. The advantages of BL are that it fosters stronger academic achievement, student engagement, and cognitive engagement and understanding as well as flexible and quick communication skills, faster interaction skills, technical skills, and adaptability to ever-changing educational practices. On the other hand, BL has brought about some pedagogical and technical difficulties for both learners and practitioners. This study found that most BL courses are not as effective as they could be because they do not have a strong pedagogical framework. Moreover, BL suffers from the technical incompetence of teachers and students, the inefficiency of LMSs, and the unavailability of required resources, such as certain devices and the Internet. Some higher education institutions have become pioneers in Chinese educational practice and been able to successfully adopt BL frameworks and integrate Moodle as well as other platforms and techniques. However, many other institutions’ attempts to adopt BL approaches have not been as effective. In order to better understand how and in what ways BL is being integrated into the educational system, this study overviews the current situation and discusses the strengths and weaknesses of BL in Chinese higher education.

## Introduction

Blended learning (BL) is the practice of teaching and learning through both online and offline models in a way that is equally distributed; meaning half of the lesson is taught face-to-face (f2f) and the other half takes place virtually (Garrison and Vaughan, 2008; Yang et al., 2019). This approach uses online and offline methods (synchronous and asynchronous) to run teaching–learning activities (Lee et al., 2016) and is meant to complement f2f learning activities (Garrison and Vaughan, 2008). As the term itself implies, BL requires a mixture of traditional practices (i.e., those that take place in the classroom with physical interactions among students and teachers) and virtual activities (i.e., online classes that use online resources) (Wang, 2021). In summary, BL combines f2f classroom interactions and computer-assisted systems and creates classes with a well-crafted portion of physical presence and virtual performances (Graham et al., 2013). Garrison and Kanuka (2004) define BL as “a thoughtful integration of classroom f2f learning experiences with online experiences.” Furthermore, attempts to combine f2f and technology-assisted forms of instruction also fall under the category of BL (Porter et al., 2014).

Because BL plays different roles depending on the context, it is very difficult to define its concrete meaning and scope (Dziuban et al., 2006; Siemens et al., 2015; Medina, 2018). Instead, the definition of BL has been extended to many other techniques and practices that take place in contemporary educational settings. However, because the meaning and scope of BL are widening at such a fast pace, it is important to identify some of its defining characteristics. In short, BL refers to a mixture of online and in-person delivery methods in which a virtual dynamic supplants many f2f classroom activities instead of merely complementing the lessons (Siemens et al., 2015). BL has also been called hybrid learning, online learning (OL), technology-mediated learning, and distributed learning (Graham et al., 2013; Siemens et al., 2015).

A variety of techniques and tools have been introduced since the 2000s (Rasheed et al., 2020) and are still being developed every day in educational settings and landscapes worldwide (Serdyukov, 2017). BL is one of the newly introduced educational techniques (Rao and Science, 2019) that have been adopted in educational settings over the last few decades (Mims-Word, 2016). China, in particular, has drastically upgraded the settings of higher education institutions by shifting from traditional methods to the next generation of educational practices (Fan, 2020). Chinese universities and colleges have been able to incorporate many of the latest techniques, pedagogic frameworks, and technologies to revolutionize their quality of education (Lee and Yuan, 2018). With the development of certain technologies, many innovative techniques have been applied to the teaching–learning practice. BL is one of the most promising new techniques that integrates online and offline activities into a combined form of teaching, and it has recently come to be viewed as an effective solution to the problems posed by educational practices that are either strictly online or in-person (Fan, 2020).

As China has worked to develop the higher education landscape through the integration of the latest educational frameworks and adoption of different technologies, such as BL models and big data, it has also welcomed a number of projects aimed at introducing BL in educational institutions throughout the entire country (Zhu, 2019; Fan, 2020). Chinese higher education sectors have been equipped with the most promising and latest frameworks (Rui, 2014), and BL has been employed as a tool to help facilitate the adoption of these models and transform the higher education sector for the next generation (Lim et al., 2019). Institutions for higher learning have been trying to make educational practices more dynamic, and because of this, how to go about transforming higher education in China is a question that has been widely studied. Therefore, determining the effectiveness of BL in higher education settings is an important issue for policymakers. The current study is thus expected to serve an important role in providing insight for researchers who are involved in the integration of BL in Chinese higher education.

## Methodology

The current systematic review was conducted based on the suggestions offered by Page et al. (2021) and Liberati et al. (2009). Previous systematic reviews (Alammary, 2019) that adopted a similar scope were also consulted while designing this review. First, we finalized the research questions and then created a protocol for finding the relevant articles. We believed this was a necessary step to minimize the possibility of research bias (Alammary, 2019). The research protocol used in this review included designing research questions and creating a search strategy for article collection; criteria for the inclusion and exclusion of studies; a selection of scientific databases; and an approach for selection, screening, extraction, and analysis.

### Research Questions

This study aimed to answer the following research questions:

1. What pedagogical frameworks and technical methods have been applied in BL practices in Chinese higher education settings?
2. What advantages and prospects has BL already provided in Chinese higher education?
3. What challenges and difficulties have emerged around the implementation of BL in Chinese higher education practices?

### Inclusion and Exclusion Criteria for Studies

This study followed a set of predetermined criteria for the inclusion and exclusion of articles. In order to determine which studies would be included in the systematic review, we used the following criteria:

1. The study used BL as a teaching method, which means that both f2f and online components were present.
2. The study was conducted in a Chinese higher education setting, which means that the study included research on courses that used BL as teaching tool in Chinese universities.
3. The study was published in English.
4. The article about the study was available online.

The following exclusion criteria were applied during the selection process:

1. The study did not use a proper BL approach.
2. The study was conducted in a non-Chinese higher education setting.
3. The study was written in a language other than English.
4. The full article about the study was not available online.

### Search Strategy and Selection of Relevant Literature

Searching for appropriate literature is the most important step of a systematic review. This study thus viewed the literature search as a significant task that involved drawing out the most relevant and meaningful pieces of scholarship. We searched reliable databases that are closely linked to advanced learning technologies and pedagogies: EBSCO, Springer Journals, Taylor & Francis, ProQuest, ScienceDirect, Eric, and SAGE Journals.

These databases are all highly regarded and include leading publications on educational and pedagogical innovations, and because of this, they are considered to be reliable for surveying the latest research in these fields. The literature search began in March 2021 and concluded in April 2021. We used different strings in each of the databases while conducting this literature review because each platform uses a distinct set of algorithms, channels, styles, and search systems. However, all of the strings that we used were subtly connected and similar to the keywords that appear in the title of this study.

Apart from the query strings mentioned above, this study used many other terms, phrases, and lexical resources that share the same or very similar meanings with the strings presented in Table 1. In addition to “blended learning,” other terms such as “flipped learning” were used. Instead of “higher education,” the term “universities” was adopted while searching for related works and widening the scope of the literature selection. Terms such as BL, hybrid learning, and flipped and OL were sometimes used interchangeably in order to extend the search and open up new resources. The search covered titles, abstracts, authors, and keywords to minimize the appearance of irrelevant articles.

**TABLE 1:** Applied query strings.

Database	Used query strings
EBSCO	Blended learning in Chinese higher education AND blended learning in China AND hybrid learning in China AND prospects and challenges
Springer Journals	Blended AND learning AND in AND Chinese AND universities AND “flip learning in Chinese higher education” AND (blended OR learning OR in OR China)
Taylor & Francis	Blended learning in Chinese higher educational settings
ProQuest	Blended learning in Chinese universities AND prospects AND challenges AND future
ScienceDirect	Blended learning in Chinese universities: prospects, challenges, solutions, future
Eric	Blended learning in Chinese higher education AND flip learning in Chinese universities AND hybrid learning in Chinese universities AND blended learning in China
SAGE Journals	Blended learning in Chinese higher education AND flip learning in Chinese universities AND hybrid learning in Chinese universities AND blended learning in China

After the literature search, all of the studies were exported to the referencing tool (i.e., Mendeley) in order to perform the data check and remove any duplicate studies. After removing the duplicates, we screened the titles and abstracts using the inclusion and exclusion criteria. In cases where the authors did not agree on whether to include or exclude a study, the full paper was read to make a decision. Two independent reviewers were also invited to check and confirm the whole screening process.

### Data Extraction

After the selection process, the data was extracted from the collected studies using the data extraction form. The data extraction form was developed specifically for this review and tested on a sample of five papers (Table 2).

**TABLE 2:** The items included on the data extraction form.

Data extraction items	Description
Title	Title of the paper
Author(s)	Names of all the authors included in the study
Publication date	The date when article was published (from January 2016 to April 2021)
Type	The type of paper (i.e., journal article, conference proceedings, report, etc.)
Applications	Tools and components of blended learning used in the course
Benefits	The positive effects of the blended learning approach
Challenges	The difficulties/challenges/reactions of the blended learning approach
Comments and future	Remarks on the quality of paper and comments about future work

### Quality Assessment of Articles

In order to assess the quality of the studies included in the review, we used the quality assessment tool developed by Rowe et al. (2012) and applied by Alammary (2019). It is a quantitative, qualitative, and mixed-method critical appraisal tool used to assess the quality of the key characteristics of an article (i.e., theoretical background, study design, data collection, data analysis, interpretations, and conclusions). Each characteristic received a score of 1 if it met the quality criteria or a score of 0 if it did not meet the quality criteria. The quality assessment was performed by the authors and reviewed by the two independent reviewers. Initially, all of authors worked independently to score the items of each article included in the study. Afterward, two reviewers were asked to score all the items included in the quality assessment criteria. All of the results from the authors and reviewers were discussed until an agreement was reached. It is important to note that the quality assessment was primarily designed to provide a deeper understanding of the different features of the studies, and no study was excluded based on the assessment.

### Data Analysis

After completing all of these steps, the data was analyzed by focusing on predetermined main themes that evolved from the research questions. The recurrent and major themes that were integrated into the data analysis were connected to BL applications, the advantages and challenges of BL, and recommendations for future development. In addition to these main themes, several related and minor themes were also taken into consideration and analyzed.

## Results

**FIGURE 1:**
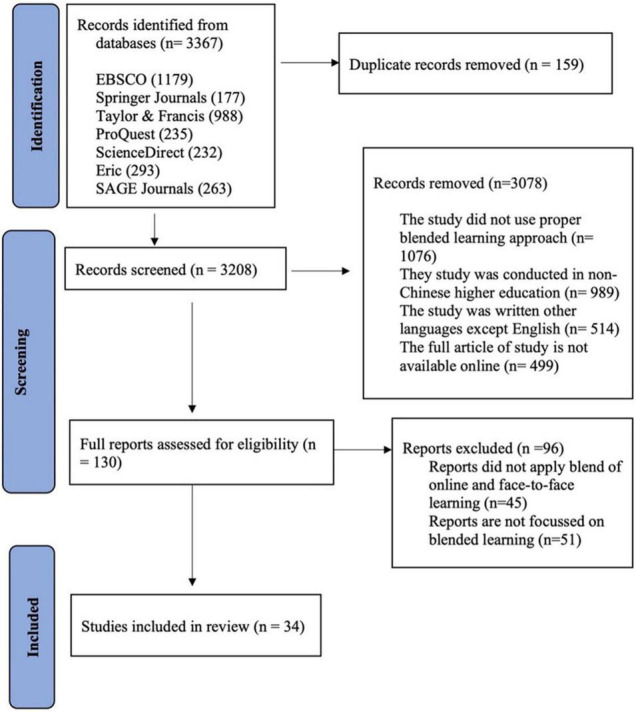
Flowchart of the systematic search of the literature.

Our initial search of the seven leading databases in educational science resulted in the collection of 3,367 articles. After accounting for duplication, 159 articles were removed. At this point, articles were screened according to their titles and abstracts, which resulted in the removal of 3,078 articles. The full text of the remaining 130 articles was accessed, and 96 articles were removed. In total, 34 articles were selected for the study based on the exclusion and inclusion criteria (see Figure 1).

### Publication Year

**FIGURE 2:**
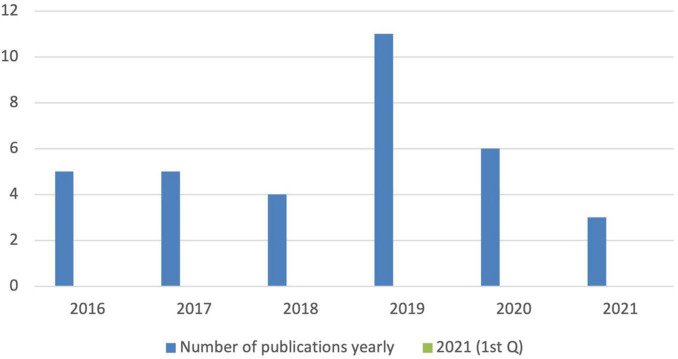
The yearly distribution of the publications included in this study (2016–2021*). *The current study included articles that were published between January 2016 and April 2021.

Among the 34 articles selected for systematic review, 5 articles were published in 2016, 5 were published in 2017, 4 were published in 2018, 11 were published in 2019, 6 were published in 2020, and 3 were published in the first quarter of 2021 (Figure 2). In 2016, 2017, and 2018, there were a substantial number of research outputs on BL in Chinese higher education. However, in 2019, research on BL grew dramatically and accounted for the highest number of scholarly publications in China. However, 2020 saw a significant decrease from 2019 with respect to the number of scholarly publications in this area, which was likely due to the global coronavirus disease 2019 (COVID-19) pandemic.

### Quality Assessment

The quality assessment of the included articles was performed using the quality appraisal tool, which consisted of five items. All of the included studies showed good quality (see Table 3). Among the 34 studies, 20 studies received a score of 5, which means that they met all of the five quality appraisal criteria. Six studies met four out of five criteria, and seven received a score of 3. Seven studies had a score of 3, while only one showed severe quality issues with a score of 2 out of 5.

**TABLE 3:** Summary of quality assessment by criterion.

Criterion	Quality of assessment of studies	
	Met criterion	Did not meet
Outcome measures	28	06
Background/literature review	26	08
Sample	29	05
Study design/methodology	30	04
Conclusion	32	02

Furthermore, the item of background/literature review was the most unmet criteria, with eight studies failing to meet it. The outcome measures was the second most unmet criteria, with six studies being unable to meet it. Among the other three criteria, five studies had quality issues related to samples, and four studies showed quality issues related to study design. Only two studies had quality issues related to conclusions (see Table 3).

### Frequently Used Techniques for Applying Blended Learning in Chinese Higher Education

Blended learning is an emerging and innovative pedagogical advancement in education (Siemens et al., 2015), and there are many innovative ways to design and apply this approach in practice (Soler et al., 2017). BL practitioners need to use a certain framework and a learning management system (LMS) or online platform that aligns with certain pedagogy requirements and teaching environments. This study found that most practitioners used various types of techniques, frameworks, platforms, and online resources while crafting BL courses to serve their teaching goals. We identified nine categories of technical and pedagogical methods that have been applied in the selected studies (Table 4).

**TABLE 4:** Frequently applied blended learning (BL) techniques and methods in Chinese higher education.

Frequently used techniques in BL	Articles
Blended course(s) with a certain applied framework or model	Huang, 2019; Yang et al., 2019, 2021; Zhang J. H. et al., 2020; Zhang Z. et al., 2020; Shi et al., 2021
Specific tools or techniques [integrated with official LMS (i.e., Moodle)]	Lai et al., 2016; Lee et al., 2016; Luo et al., 2017; Lu et al., 2018; Teo et al., 2019; Li X. et al., 2020
Digital platforms offered in the course(s) other than the official LMS	Shu and Gu, 2018; Wang et al., 2019; Yao 2019b; Gao et al., 2020; Li L. et al., 2020; Li X. et al., 2020; Wang, 2021
Blended courses	Huang, 2016; Lai et al., 2016; Sun and Qiu, 2017; Tsai et al., 2018; Chan, 2019; Law et al., 2019
Unspecified category of tools or platforms used in BL	Li et al., 2019; Yang et al., 2021
Digital storytelling and gamification (online platform)	Yao (2019b)
Social media and other apps (Mindomo, Poll Everywhere, WeChat, Zoom)	Zhou and Li, 2019
Workshop	Ding et al., 2017
SECI model with Google services (Plus, Drive, Blogger, and Sites)	Jou et al., 2016

Among the 34 studies, 7 articles [the highest number of articles (20.58%)] showed that teachers use “digital platforms in the course(s)” other than the official LMS. Six articles (17.64%) reported that teachers apply “specific tools or techniques [integrated with official LMS (i.e., Moodle)].” Another group of six articles (17.64%) reported that teachers use “blended course(s) with a certain applied framework or model” in order to benefit their teaching. Six articles (17.64%) indicated that blended courses are used in the adoption of a hybrid learning model.

Other than the aforementioned applications of techniques, systems, and models, some other methods and techniques have been used in the application of BL. Two articles (5.88%) showed no specific use of Moodle of LMS in the practical application of hybrid learning. Another article reported “digital storytelling and gamification (online platform)” as types of BL platforms. One article (2.94%) reported the use of social media apps (e.g., Mindomo, Poll Everywhere, WeChat, and Zoom). Another article used the SECI model with Google services (i.e., Plus, Drive, Blogger, and Sites) in order to train students and teachers and improve the quality of higher education.

### Advantages of Blended Learning in Chinese Higher Education

As shown in Table 5, the advantages of BL have emerged in Chinese higher education in different ways over the past several years. BL has numerous benefits, which have been divided into 16 specific categories: fostering learning and academic achievement, promoting stronger cognitive engagement and interaction, encouraging student autonomy and confidence, allowing for flexible and convenient learning environments, kindling learning motivation, fostering positive attitudes and active learning behaviors, improving self-respect and satisfaction, raising performance levels in EFL/ESL (English language learning) courses, providing opportunities for learner-centered and personalized learning, creating new skills and resources, offering quick and effective feedback, bridging the gap between advantaged and disadvantaged groups of learners, improving critical thinking capacity, developing academic help and sharing skills, sparking knowledge transformation, and overcoming learning anxiety through the adoption of self-learning strategies.

**TABLE 5:** The advantages of BL in Chinese higher education from this synthesis.

Benefits of blended learning	Articles
Learning and academic achievement	Luo et al., 2017; Yao, 2017, 2019b; Law et al., 2019; Li et al., 2019; Li L. et al., 2020; Wang et al., 2019; Yang et al., 2019; Zhang J. H. et al., 2020; Wang, 2021
Better cognitive engagement and interaction	Jou et al., 2016; Lee et al., 2016; Szeto and Cheng, 2016; Shu and Gu, 2018; Law et al., 2019; Wang et al., 2019; Yao, 2019a; Li X. et al., 2020; Shi et al., 2021
Developing learners’ autonomy and confidence	Lai et al., 2016; Lee et al., 2016; Yao, 2017, 2019b; Wang et al., 2019; Zhang Z. et al., 2020
Flexible and convenient learning environment	Jou et al., 2016; Sun and Qiu, 2017; Yao, 2017, 2019a,2019b; Wang et al., 2019; Zhou and Li, 2019
Learning motivation	Jou et al., 2016; Lee et al., 2016; Teo et al., 2019; Wang et al., 2019; Li X. et al., 2020; Zhang J. H. et al., 2020; Shi et al., 2021; Wang, 2021
Positive attitude and active learning behaviors	Tsai et al., 2018; Zhang J. H. et al., 2020; Zhang Y. et al., 2020; Zhang Z. et al., 2020
Developing self-respect and satisfaction	Li et al., 2019; Li L. et al., 2020; Wang et al., 2019; Li X. et al., 2020; Zhang Y. et al., 2020
Better performance in EFL/ESL course (English language learning)	Huang, 2016; Sun and Qiu, 2017; Yao, 2017, 2019a; Wang et al., 2019; Wang, 2021
Learner-centered and personalized learning	Wang et al., 2019; Yao, 2019a; Xing, 2020; Zhang Y. et al., 2020
Developing resources and new skills	Jou et al., 2016; Lai et al., 2016; Xing, 2020; Zhang Y. et al., 2020
Quick and effective feedback	Luo et al., 2017; Sun and Qiu, 2017; Yao, 2019b
Bridging gaps between advantaged and disadvantaged groups of learners	Yang et al., 2019; Yao 2019a
Improving critical thinking capacity	Jou et al., 2016; Xing, 2020
Developing academic help and sharing skills	Yao, 2017, 2019b
Knowledge transformation	Jou et al., 2016
Overcoming learning anxiety through the adoption of self-learning strategies	Yao, 2017, 2019b

Although the application of BL in Chinese higher education has a range of benefits, several major advantages emerged during our analysis of the literature. Most significantly, out of a total of 34 studies, 10 (29.41%) showed that BL benefited teachers and learners by fostering learning and academic achievement. Similarly, nine of the articles (26.47%) included in this study indicated that BL clearly improves cognitive engagement and interaction. BL has also been found to develop learning motivation among learners. Eight (23.52%) articles demonstrated this particular benefit of BL in Chinese higher education. The fourth most useful application of BL is related to offering and arranging a “flexible and convenient learning environment” for students who learn through BL platforms. Seven articles support the idea that BL systems offer an outstanding opportunity to provide learners with a flexible learning environment. The fifth benefit of BL is that it allows learners to develop their autonomy and confidence in higher education in the Chinese context. Six articles (17.64%) clearly indicated this advantage while studying BL courses in Chinese educational settings.

Blended learning has also impacted Chinese higher education by improving the attitudes and learning behaviors, developing self-respect and satisfaction, fostering better performance in EFL (English as a foreign language)/ESL (English as a second language) courses (English language learning), offering opportunities for learner-centered education and personalized learning, and sparking the creation of new resources and skills. Six (17.64%) articles discussed the role and advantages of BL in foreign language teaching and learning activities. Researchers working in this field have concluded that BL benefits foreign language learning in Chinese higher education environments. Five articles (14.70%) revealed that BL has a positive impact on developing the self-respect and satisfaction of learners. The remaining three benefit categories (positive attitude and active learning behaviors, learner-centered and personalized learning, and new resources and skills) were demonstrated in four (11.7%) of the articles.

Three articles (8.82%) reported that BL offered a “quick and effective feedback” system between teachers and learners that lessened the gap between these two major stakeholders. Two (5.88%) of the studies found that BL bridges the gap between advantaged and disadvantaged groups of learners from the city and rural areas in the context of Chinese higher education. Another category, “improving critical thinking capacity,” was supported by two articles (5.88%). Two articles (5.88%) also supported the claim that BL allows students to receive more help and share their skills, and two articles (5.88%) reported that BL allows learners to overcome learning anxiety through the adoption of self-learning strategies. Two articles (5.88%) reported the benefit of BL for the learners overcoming the learning anxiety and self-learning strategies. Only one article supported the idea that BL promotes knowledge transformation by allowing for flexibility without having to face the difficulties of different learning boundaries and other lagged systems.

### Challenges of Blended Learning

As shown in Table 6, there are many challenges to applying BL in Chinese higher education that have yet to be solved, which means that the benefits of BL are yet to be fully harnessed. These challenges have been divided into 12 major categories. The shortcomings of BL include a lack of a sound pedagogical and instructional design; a lack of digital literacy and training for using BL; a lack of student autonomy and competencies; a lack of techniques, devices, tools, and infrastructure; extra workload; a tendency to be time-consuming for the teachers; an emphasis on dominance and difference between different groups of learners; technical problems; biasedness of tools and systems; an insufficient scope of interaction and interpersonal communication; a lack of access to the Internet; difficulty engaging learners; and clashes with Chinese culture, practices, and educational standards.

**TABLE 6:** The challenges and drawbacks of blended learning in Chinese higher education.

Challenges of blended learning	Articles
Lack of sound pedagogical and instructional design	Huang, 2016, 2019; Lai et al., 2016; Lee et al., 2016; Sun and Qiu, 2017; Yang et al., 2019; Zhou and Li, 2019; Li X. et al., 2020; Zhang Z. et al., 2020; Shi et al., 2021; Wang, 2021
Lack of digital literacy and training for using BL	Jou et al., 2016; Teo et al., 2019; Zhou and Li, 2019; Wang, 2021
Lack of student autonomy and competencies	Sun and Qiu, 2017; Teo et al., 2019; Wang et al., 2019; Yang et al., 2019
Lack of techniques, devices, tools, and infrastructure	Yang et al., 2019; Zhou and Li, 2019; Zhang Z. et al., 2020
Extra-workload and time-consuming for the teachers	Lai et al., 2016; Sun and Qiu, 2017; Huang, 2019
Clashes with Chinese educational standards, practices, and culture	Ding et al., 2017; Wang and Han, 2017; Chan, 2019
Making learners engaged	Lai et al., 2016; Shi et al., 2021
Dominance and difference between different groups of learners	Yao, 2017; Wang et al., 2019
Technical problems	Li X. et al., 2020; Wang, 2021
Biasedness of tools and systems	Zhang Z. et al., 2020
Insufficient scope of interaction and interpersonal communication	Chan, 2019
Lack of access to the Internet	Yao (2019a)

Among the drawbacks of BL, the challenge of developing sound pedagogical and instructional designs is the issue mentioned the most frequently in the selected studies. Eleven (32.35%) articles reported this problem. Four articles (11.76%) reported a “lack of digital literacy and training for using BL” as the second most frequently faced problem among teachers and students. Four articles (11.76%) found that a “lack of student autonomy and competencies” is another challenge of applying and operating BL in Chinese higher educational settings. Although BL promotes self-reliance and autonomous learner competencies, there are still passive learners and technically less efficient learners who cannot adopt BL practices. The fourth highest reported (11.76%) BL challenge category is related to the techniques, tools, and infrastructure required to apply BL in practice in Chinese higher education settings.

Three articles (8.82%) reported that BL has created an extra workload for teachers who have been burdened with extra work, such as organizing traditional classrooms while arranging and authoring the virtual component of BL courses on different platforms. Similarly, another three articles (8.82%) reported that BL clashes with Chinese culture, practices, and educational standards. Two articles (5.88%) reported that some learners exert dominance over other students in blended classes. Another two (5.88%) also found that engaging learners in the learning process during online teaching modules is very challenging, as many learners are often passive and reluctant to engage in the learning process.

Two articles reported technical problems, and the remaining three categories of challenges have each been reported in the selected studies. One article (2.94%) reported that the biasedness of tools and systems often occurs in the teaching–learning process while practicing BL. An insufficient scope of interaction and interpersonal communication (2.94%) has also been identified as a challenge in BL. Finally, an article reported that poor or unavailable access to the Internet is one of the problems that emerge while attempting to apply BL in Chinese higher education environments.

## Discussion

The surveyed articles indicated that research on BL was not very trendy in Chinese higher education in 2016. However, studies on this form of teaching started to surge later in the year and continued to grow in number from that point onward. In 2019, 11 studies were published on BL and accounted for the highest number of scholarly publications in China on the developments in technological forms of “pedagogical advancement.” Our survey of the selected 34 articles allowed us to answer the research questions that were established at the beginning of the study. The following discussion section overviews our findings and is organized around each of the three major research questions.

### What Pedagogical Frameworks and Technical Methods Have Been Applied in Blended Learning Practices in Chinese Higher Education Settings?

Blended learning is highly associated with the transformation of technologies and the pedagogical framework, pedagogical practice, and embedded systems of educational institutions. Because technology and innovation in education are ever-changing, BL must also cope with these types of changes. There are many frameworks and techniques for applying BL that have been adopted in Chinese higher education, the most used of which include Moodle, multi-functional frameworks, LMS, digital platforms, blended courses, and many other officials and privately operated technical and pedagogical methods.

Using “digital platforms that are offered in the course(s) through a means other than the office LMS” is the technique that teachers apply the most frequently while practicing BL in Chinese higher education environments. Some tertiary educational institutions have developed an official LMS or use Moodle, but many other tertiary educational institutions have neither adopted nor developed any official LMS to take full advantage of hybrid teaching and innovate learning practices (Yao, 2017, 2019a; Han et al., 2019). As a result, many teachers used the platforms that they believe are best suited to their purposes. They pick certain platforms from the many communication apps, such as Zoom, for delivering their teaching methods in a way that might be compatible enough with the online component of hybrid learning. Seven articles from the current study identified the problem of applying platforms other than the official LMS. If teachers and students had the opportunity to use the developed LMS or Moodle, they would have been able to grow in their self-efficacy, self-regulation, skills, and pedagogical usage.

The second most frequently mentioned technique that we found appeared in six studies and involved a BL framework that depended on specific techniques that serve the purpose of BL from a pedagogical and practical perspective. Teachers applied specific tools, software, frameworks, and platforms that worked as peripheral or integrated components of the official LMS (Li X. et al., 2020). These frameworks and strategies functioned as elements of the institutional LMS. However, the studies found that teachers did not often fully apply resources other than the official LMS. Moreover, some of the articles indicated that teachers used BL courses as their main tool for applying certain pedagogical frameworks into their teaching practice. In the literature, the adoption of these types of courses is thus presented as not being compatible with the goal of fulfilling pedagogical and technical requirements, which means that they might not provide the best education to learners.

The remaining studies utilized whatever other technical and pedagogical methods that they deemed appropriate for serving the course material and lesson. One of the studies applied a platform that uses digital gamification (Yao, 2019b), and another used social media (i.e., Mindomo, Poll Everywhere, WeChat, and Zoom) as the sole platform for applying BL pedagogical and technical features to learning activities (Zhou and Li, 2019). Another study adopted the facilities of the SECI model with Google services (e.g., Plus, Drive, Blogger, and Sites) in order to help students learn in more open and more targeted ways (Jou et al., 2016). Two articles clearly showed that some teachers did not use any specific framework, technique, official LMS, or other known digital platforms. This type of use has been renamed as an “unspecified category of tools or platforms used in BL” (Yang et al., 2021). This study ultimately found that most practitioners did not use any official LMS.

### What Advantages and Prospects Has Blended Learning Already Provided in Chinese Higher Education?

We found that BL has already proven to be efficient and necessary in the educational landscape of Chinese higher education (Medina, 2018). The first category that BL benefits from concerns the learning and academic achievement of the learners (Wang et al., 2019). A large proportion of the studies (29.41%) found that the learning outcome was better in BL than in traditional classroom practices with respect to performance and achievement. Moreover, these studies found that learning through BL was more cognitive than traditional classroom learning. Zhang Z. et al., 2020 proposed that BL significantly impacts habit formation and behavioral patterns and that changes in task responses allow students to not only adopt to BL but also become more adaptable to other similar type of technologies that could help facilitate their learning and skill development. As learners adopt newly emerging techniques and technologies, their level of performance improves.

Cognitive understanding and interaction also emerged as an important category in this review. Many of the selected studies found that quick and flexible interaction among peers and teachers played an important role in allowing students to engage in the learning process. This combined learning system ultimately gives learners an opportunity to develop their cognitive understanding of the lesson (Shi et al., 2021). Adopting a more flexible and convenient learning environment also allows teachers and students to interact with one another in ways that allow for more flexibility than traditional learning environments. Because of this, BL styles have been quickly accepted by both teachers and students. Fast interactions, flexible environments, and feedback that promotes personalized teaching and learning ultimately lead to cognitive understanding and learning output (Zhou and Li, 2019).

Blended learning has led to another significant development in pedagogic practice by pushing students to learn and adopt new technologies, methods, and approaches while taking blended courses. Blended courses encourage learners to become proficient in a variety of emerging software, tools, platforms, and technical skills. These skills encourage learners to develop their efficacy in practical life and the workplace (Lai et al., 2016). The selected studies reported that with BL, students learned in ways that allowed them to interact and share with their peers. In this way, quick feedback and uninterrupted communication played a significant role in giving learners the skills that they need to perform well in the practical world.

China has been subjected to an imbalanced educational infrastructure that creates a division between developed and underdeveloped regions and male and female students. Ensuring equal opportunities for education and sustainable development is a challenging task. The possibility of bridging the gap between enriched educational institutions and tertiary institutions with poor resources is one of the most visible and promising advantages of adopting BL in China. The findings in the selected articles indicated that most adult learners believe that BL can help reduce the gap between advantaged and disadvantaged learners throughout the country (Yao, 2019a). It could also allow female learners to participate in more advanced learning systems, courses, and skill development programs. Thus, discrimination will have less of an impact at the national level, and the centralization and urbanization of people and resources can be actualized.

Blended learning models also foster learning motivation better than traditional classroom practices. Zhang Y. et al., 2020 explained that BL allows students to have quick interactions with their classmates and instructors, which makes the learning process more dynamic. Fast and flexible learning styles ultimately allow students to be more autonomous and independent and become more motivated to learn. BL activities, thus, kindle positive attitudes in learners. Moreover, learners receive a timely response after completing certain tasks, and because of this, they adopt more active learning behaviors. Other related factors that have a positive impact on student motivation include personalized feedback and getting inspiration from the instructor. Flexible learning environments, positive attitudes, and active learning styles can thus reap better outcomes.

Blended learning creates self-respect and satisfaction among learners by allowing them to manage their own learning pace and use an independent approach while completing a lesson. The satisfaction that students experience from BL manifests in multiple ways. First, BL environments encourage emotional engagement and perceived usefulness. Second, BL promotes course satisfaction through emotional engagement. Emotional engagement motivates students to engage in learning in ways that make them feel more satisfied when compared to other styles of learning (Gao et al., 2020). Thus, personalized learning approaches have been linked with greater student satisfaction because they allow learners to learn at their own pace according to their individual capacity and learning level (Li L. et al., 2020; Li X. et al., 2020).

Because BL combines virtual and traditional classroom arrangements, it allows foreign language learners to use fast and flexible interaction systems that encourage them to engage in the learning process and refine their language skills in everyday life through virtual platforms that are designed for BL courses (Yao, 2019a). Hybrid learning allows for flexible communication and feedback opportunities, which leads to the quick and efficient application of foreign language learning techniques (Wang et al., 2019). Thus, many studies found that foreign language learners perform better in BL settings than they do in traditional classrooms. BL allows learners to become engaged and autonomous and personalize the learning process, which makes them more self-dependent and active (Castaneda, 2016).

Finally, other benefits include improving critical thinking, developing learning strategies, and allowing for academic sharing and knowledge transformation. The spread of knowledge is expected to broaden across the nation, which should allow the knowledge gap to close in the future. Fast and multifunctional learning approaches also make learners more engaged, interactive, and motivated. As a result, their critical thinking capacity becomes more active and improves when they participate in BL. Knowledge construction and transformation are tasks that can be improved, shared, and disseminated through academic communication and interaction that occur quickly through BL. Finally, students learn how to develop their learning strategies for adopting and coping with ever-changing educational transformations and developments.

### What Challenges and Difficulties Have Emerged Around the Implementation of Blended Learning in Chinese Higher Education Practices?

There are various advantages associated with applying BL in practice, but it also poses many challenges. The most frequently faced problem linked to BL is the “lack of sound pedagogical and instructional designs.” This means that there are various online technologies, techniques, and infrastructures designed for facilitating the delivery of education and paving the way for educational transformation nationwide, but they are not entirely designed for education purposes or suited to pedagogical frameworks. BL is largely dependent on assistive technologies that require a significant restructuring of pedagogical practice. Throughout the current review, the weaknesses of pedagogical designs have been reported by many articles (11 = 32.35%), which state that BL has the potential to transform Chinese higher education if educators and learners can learn to cope with BL styles.

The difficulties of designing a BL course include both technical and pedagogical issues. Transforming lesson plans and creating content in ways that conform to digital delivery methods and LMS while aligning with pedagogical designs and theories that can help learners acquire knowledge in a progressive and self-directed manner can be challenging. BL cannot be successful unless teachers upgrade their skills and integrate the “technological content knowledge” with the “pedagogical knowledge” (Hungwe and Dagada, 2013). Another challenge is linked to how efficiently learners adapt to the course content and the flow of lesson operation (Lee et al., 2016). One study reported that many students taking BL courses face difficulties while accessing the resources and downloading them to facilitate their education (Zhang Y. et al., 2020).

A lack of digital literacy and an inability to develop BL skills are two of the most prevalent problems that emerge in BL practice. We found that 11.76% of the selected studies reported that teachers and students lack the skillset that they need to apply BL in real practice. BL requires a sound level of proficiency in digital literacy components, such as computing and handling digital tools. However, the subjects who participated in these studies showed that they did not have sufficient skills to apply BL universally. This problem is also linked to a “lack of autonomy and competency” in students and learners. If these major groups of BL stakeholders cannot adapt to these new technological and pedagogical practices, BL cannot be developed. Although many articles glorify the autonomy that students develop through BL, it can be very confusing for students to learn how to be autonomous if they have not mastered certain skills that are crucial to their ability to engage in BL practices.

Blended learning has allowed the students to learn in more individualistic and personalized ways. However, the extent to which it can offer effective individualistic instruction has been a topic of debate. BL can offer freedom of time, space, and progress while developing certain educational skills. This student-centered and personalized approach has its benefits, but no single pedagogical framework, technical model, or infrastructure has been established to deal with such a large number of learners at once. Moreover, personalized interactions between teachers and students might slow down the learning process in ways that allow students to reach learning objectives. A traditional classroom allows learners to compare, contrast, and adapt to the pace of their classmates, and that, combined with the lesson plan and syllabus, helps keep students at the same pace and encourages them to make progress. However, if teaching operates in a more private way, learners might lose their motivation to learn and develop. They might become distracted, disconnected, frustrated, and demotivated to progress in their studies while working in isolation.

Blended learning requires the adoption of various kinds of tools, techniques, devices, and skills that many of the learners cannot use. For example, there are many learners who do not have powerful devices, tools, Internet connection, or proficiency in the operation of these resources. There are also many students who feel more engaged when they are in the physical presence of their classmates compared to when they connect over online platforms. Moreover, there are very few frameworks and online infrastructures that can serve the purpose of teaching, learning, transforming, adapting, and spreading BL in practice in Chinese higher education. The majority of the articles in this study reported that most frameworks and infrastructures are still not highly developed or widely used in mainstream teaching practices. BL, thus, has a long way to go before it can replace traditional classroom practices. Finally, there are many students who do not have access to the high-speed Internet connections that are essential to BL practice. This is one of the biggest challenges of adopting BL in Chinese educational practices, especially in rural areas.

Blended learning requires teachers to establish a certain ratio between online and f2f teaching practices. Because the same teacher needs to arrange both the offline and online components of the teaching schedule, materials, frameworks, and other relevant protocols, they end up investing a lot of time and effort in the preparation, organizations, and communication of the different modules. Teachers who use BL methods thus often do twice as much work compared to instructors who only use traditional classroom management practices. This ultimately works against the interest of teachers and the overall objectives of BL learning, which seek to reduce the labor and cost of practitioners and learners while enhancing quality education in Chinese higher education. Moreover, Chinese educational practice has a strong culture of close cooperation between teachers and students that is shaped by the values of virtue, diligence, and obedience. Chinese educational practice has a long tradition of authority that is interchangeably interpreted as a close relationship or mentorship between teachers and students. In this sense, these cultural characteristics go against the core values of BL (Chan, 2019).

Finally, there are some other challenges that need to be dealt with if BL is expected to be successful in Chinese higher education. Biasedness of tools and systems can occur in any system if the course instructors, course designers, and technologists cannot make a sound framework and establish the type of infrastructure that is required for BL practices. There are many examples of instances in which the technology manipulated the educational practice. There are also many technical problems that occur when users do not have any previous experience with BL. More importantly, some dominant students take control over the whole classroom, which can spoil the opportunity to interact with the other students (Wang et al., 2019). As mentioned before, BL might prevent students from communicating and socializing with one another because BL does not allow learners to spend much time with their peers and teachers at school. Consequently, learners miss the opportunity to socialize and participate in campus life (Chan, 2019).

### Limitations of the Research

This study has been conducted very carefully to maintain all the systematic review writing protocols, standards, and regulations. This study utilized the search protocol and other regulations of the PRISMA framework and applied them throughout the whole study. However, it still might have some limitations. For example, we only selected scholarship that appeared in leading databases, such as Clarivate. Consequently, we may have overlooked some studies that were compatible with our research objectives simply because they were published in journals that were not included in the databases that we used. Moreover, we only selected the studies that were written in English. Relevant research might have not been included in this study because it was written in Chinese and thus did not meet the inclusion criteria.

Furthermore, as there are a variety of issues related to the development of different technical and non-technical resources for applying BL successfully in the educational landscape, there are differences across the included studies. These issues have been very carefully identified, grouped, and analyzed. We regret if we missed any important points or showed any biases while conducting this study. Additionally, the current study discovered that most of the included studies were conducted in the eastern areas of China. These eastern cities are highly developed and advanced in almost all sectors, including education, compared with western areas of China. Thus, the included research might not present a universal impression of Chinese educational institutions or represent how BL has been adapted nationwide.

## Conclusion and Future Directions

The introduction of BL in Chinese higher education has helped transform the educational practices. BL has been applied in many ways as a means of facilitating teachers and learners. It has undoubtedly made an impact on higher education and has already proven to be beneficial. There are many frameworks, techniques, LMSs, blended courses, online platforms, software, tools, social media, and similar platforms and tools that are currently being used in Chinese higher education. Teachers select and use whatever platforms they are interested in and feel comfortable using in their delivery. However, they are supposed to use the tools and infrastructure that has been developed to integrate BL into the LMS.

This systematic review has identified a number of opportunities for BL in Chinese higher education. There are many advantages of BL that include better academic achievement; better cognitive engagement and understanding; faster and more flexible communication skills; faster and smoother interaction skills; the development of technical skills and learner motivation; and the encouragement of autonomy, positive attitudes, active behavior patterns, satisfaction, personalized learning, critical faculty, and adaptation in students. In summary, BL allows for a student-centered approach that has long been absent in Chinese higher education. When traditional classroom practices in China started to be viewed as passive, BL brought about a drastic change that introduced student-focused learning styles and offered learners more freedom, autonomy, creativity, and agency.

China has developed many policies for transforming its educational system and taking it to the next level. Related policies are still being developed to push the nationwide education revolution forward. However, in our overview of the existing research, we found very few references to a developed nationwide LMS or Moodle that allows BL practices to be adopted widely, authentically, and flexibly. Applying this learning method in practice has also exposed many challenges. Among the many shortcomings of BL in the Chinese context, a lack of pedagogical and instructional design is the most prevalent problem that emerges while applying this hybrid learning mode in higher education environments. Most of the stakeholders and experts reported that this issue needed to be solved as quickly as possible in order to allow for BL to be used more widely. Other drawbacks include a lack of digital literacy and training; a lack of student autonomy and competencies; and a lack of techniques, devices, tools, and infrastructure. Another challenge stems from the fact that certain elements of BL contradict Chinese cultural and educational values.

Additionally, teachers face the difficulty of playing two different roles at once while planning a course. They need to design a lesson for both online and offline deliveries. This type of course design requires a huge time commitment on the part of teachers. Not only do they need to design a course in a way that meets the requirements of both online and offline learning, but they also need to create different materials for each version and offer extensive support to a large number of students. They need to lead lectures, monitor their students, participate in personal interactions, conduct assessment activities, and evaluate both versions. Instructors, thus, need to invest a large amount of time, effort, skill, and cost while using BL technologies. Moreover, Chinese educational culture has a long tradition of teacher-centered teaching practices, and educational stakeholders are used to this style. However, BL has a strong foundation in student-centered teaching, which aligns with certain elements of traditional forms of teaching in China.

Furthermore, BL has suffered from the technical incompetence of stakeholders, the inefficiency of LMS, shortages of resources, unavailability of devices, and issues with Internet connection. There is a high possibility of misuse of tools and technologies while running BL, such as the biasedness of software or tools that emerge due to faulty design or coding. Similarly, another problem exists that is related to the level of interaction and interpersonal communication that BL courses offer. Students may not have enough opportunities to interact with their peers in a physical environment, which might cause them to feel frustrated and isolated. Engaging students in the learning process, thus, becomes more complicated in BL settings when compared to traditional classroom environments. Ultimately, this study presents the current situation of BL learning in Chinese higher education and presents both its strengths and weaknesses.

## Data Availability Statement

The original contributions presented in the study are included in the article. Further inquiries can be directed to the corresponding author.

## Author Contributions

SM and MA: conceptualization and write original draft. SM: formal search for literature. MA, SM, SP, and NS: methodology. NS and SP: quality assessment. MA, NS, and SP: review of draft and editing. MA: funding acquisition and project supervision. LN: revision and proofreading. All authors have read and agreed to the published version of the manuscript.

## Conflict of Interest

The authors declare that the research was conducted in the absence of any commercial or financial relationships that could be construed as a potential conflict of interest.

## Publisher’s Note

All claims expressed in this article are solely those of the authors and do not necessarily represent those of their affiliated organizations, or those of the publisher, the editors and the reviewers. Any product that may be evaluated in this article, or claim that may be made by its manufacturer, is not guaranteed or endorsed by the publisher.
