# Supplementary material for: Pedagogical Applications, Prospects, and Challenges of Blended Learning in Chinese Higher Education: A Systematic Review
Source: Front Psychol. 2022 Jan 25;12:772322. doi: 10.3389/fpsyg.2021.772322 (PMC8821963; doi:10.3389/fpsyg.2021.772322)
Supplement: Supplementary file 1 [file Table_1.DOCX]

**S1 Table**. **Evaluation of studies quality.** √ =Criterion met; **×**= Criterion not met

|  | **Outcome measures** | **Background/literature review** | **Sample** | **Study design or methodology** | **Conclusions** | **Overall quality score** |
| --- | --- | --- | --- | --- | --- | --- |
| Yao, C. (2019) | √ | √ | √ | √ | √ | 5 |
| J. H. Zhang et al., (2020) | √ | √ | √ | √ | √ | 5 |
| Chunlin Yao (2019) | √ | √ | √ | √ | √ | 5 |
| Xiuhan Li, et al. (2020) | √ | √ | √ | √ | √ | 5 |
| Chunlin Yao (2017) | √ | √ | √ | √ | √ | 5 |
| Lu et al., (2018) | √ | √ | √ | √ | √ | 5 |
| Chan, E. Y. (2019) | √ | √ | √ | √ | √ | 5 |
| Luo et al., (2017) | √ | √ | √ | √ | √ | 5 |
| Lee, Lau, & Yip (2016) | √ | √ | √ | √ | √ | 5 |
| Na Wang, et al. (2019) | √ | √ | √ | √ | √ | 5 |
| Zhou & Li (2019) | √ | √ | √ | √ | √ | 5 |
| Huang (2019) | √ | √ | √ | √ | √ | 5 |
| Lai, Lam, & Lim (2016) | **×** | **×** | √ | √ | √ | 3 |
| Shu & Gu (2018) | √ | √ | √ | √ | √ | 5 |
| Sun & Qiu (2017) | √ | √ | √ | √ | **×** | 4 |
| Jou, Lin, & Wu (2016) | **×** | √ | √ | √ | √ | 4 |
| Wang (2021) | √ | √ | √ | √ | √ | 5 |
| Jou et al., (2016) | √ | √ | √ | √ | √ | 5 |
| Teo, Zhou, Fan, & Huang (2019) | √ | √ | √ | √ | √ | 5 |
| Y. Zhang, Chen, & Wang, (2020) | **×** | √ | √ | √ | √ | 4 |
| Zhang, Cao, Shu, & Liu (2020) | **×** | **×** | √ | √ | √ | 3 |
| Shi, Tong, & Long (2021) | √ | √ | √ | √ | **×** | 4 |
| Y. Wang & Han (2017) | √ | **×** | **×** | √ | √ | 3 |
| Huang (2016) | **×** | √ | √ | √ | √ | 4 |
| Tsai1, Chao, Lin, & Cheng (2018) | √ | √ | √ | √ | √ | 5 |
| Yang, Zhou, & Hu (2021) | √ | √ | √ | √ | √ | 5 |
| Law, Geng, & Li (2019) | √ | √ | √ | **×** | √ | 4 |
| Ding, Gao, & Lu (2017) | √ | **×** | **×** | **×** | √ | 3 |
| Gao, Jiang, & Tang (2020) | √ | √ | √ | √ | √ | 5 |
| Li, He, Yuan, Chen, & Sun (2019) | **×** | **×** | **×** | √ | √ | 3 |
| Han, Wang, & Jiang (2019) | √ | √ | √ | √ | √ | 5 |
| Szeto & Cheng (2014) | √ | **×** | √ | **×** | √ | 3 |
| X. Yang et al. (2020) | √ | **×** | **×** | **×** | √ | 2 |
| J. Yang et al., (2019) | √ | **×** | **×** | √ | √ | 3 |

**Supplementary materials**

Chan, E. Y. M. (2019). Blended learning dilemma: Teacher education in the confucian heritage culture. *Australian Journal of Teacher Education*, *44*(1), 36–51. https://doi.org/10.14221/ajte.2018v44n1.3

Ding, Y., Gao, Y., & Lu, F. (2017). The Development of QM-Fudan Higher Education Online Course Quality Standards: Some Results and Analysis. *American Journal of Distance Education*, *31*(3), 198–206. https://doi.org/10.1080/08923647.2017.1301142

Gao, B. W., Jiang, J., & Tang, Y. (2020). The effect of blended learning platform and engagement on students’ satisfaction—— the case from the tourism management teaching. *Journal of Hospitality, Leisure, Sport and Tourism Education*, *27*(September), 100272. https://doi.org/10.1016/j.jhlste.2020.100272

Han, X., Wang, Y., & Jiang, L. (2019). Towards a framework for an institution-wide quantitative assessment of teachers’ online participation in blended learning implementation. *Internet and Higher Education*, *42*(November 2018), 1–12. https://doi.org/10.1016/j.iheduc.2019.03.003

Huang, Q. (2016). Learners’ Perceptions of Blended Learning and the Roles and Interaction of f2f and Online Learning - Linguistics and Language Behavior Abstracts (LLBA) - ProQuest. *ORTESOL Journal*, *33*(2013), 14–33. Retrieved from https://search-proquest-com.ezproxy.lib.monash.edu.au/llba/docview/1942218954/6676EC21135B4CEEPQ/6?accountid=12528

Huang, Q. (2019). Comparing teacher’s roles of F2f learning and online learning in a blended English course. *Computer Assisted Language Learning*, *32*(3), 190–209. https://doi.org/10.1080/09588221.2018.1540434

Jou, M., Lin, Y. T., & Wu, D. W. (2016). Effect of a blended learning environment on student critical thinking and knowledge transformation. *Interactive Learning Environments*, *24*(6), 1131–1147. https://doi.org/10.1080/10494820.2014.961485

Lai, M., Lam, K. M., & Lim, C. P. (2016). Design principles for the blend in blended learning: A collective case study. *Teaching in Higher Education*, *21*(6), 716–729. https://doi.org/10.1080/13562517.2016.1183611

Law, K. M. Y., Geng, S., & Li, T. (2019). Student enrollment, motivation and learning performance in a blended learning environment: The mediating effects of social, teaching, and cognitive presence. *Computers and Education*, *136*(September 2018), 1–12. https://doi.org/10.1016/j.compedu.2019.02.021

Lee, Y. C., Lau, K., & Yip, V. W. Y. (2016). Blended learning for building student-teachers’ capacity to learn and teach science-related interdisciplinary subjects. *Asian Association of Open Universities Journal*, *11*(2), 166–181. https://doi.org/10.1108/aaouj-09-2016-0029

Li, C., He, J., Yuan, C., Chen, B., & Sun, Z. (2019). The effects of blended learning on knowledge, skills, and satisfaction in nursing students: A meta-analysis. *Nurse Education Today*, *82*(June), 51–57. https://doi.org/10.1016/j.nedt.2019.08.004

Li, L., Tam, C. W., Wang, N., Cheung, F., Zhou, Q., Zhang, C., … Feng, Y. (2020). Effectiveness of blending E-learning with field trip on Chinese herbal medicine education: Quasi-experimental study. *BMC Complementary Medicine and Therapies*, *20*(1), 1–9. https://doi.org/10.1186/s12906-020-03034-y

Li, X., Yang, Y., Chu, S. K. W., Zainuddin, Z., & Zhang, Y. (2020). Applying blended synchronous teaching and learning for flexible learning in higher education: an action research study at a university in Hong Kong. *Asia Pacific Journal of Education*, *00*(00), 1–17. https://doi.org/10.1080/02188791.2020.1766417

Lu, O. H. T., Huang, A. Y. Q., Huang, J. C. H., Lin, A. J. Q., Ogata, H., & Yang, S. J. H. (2018). Applying learning analytics for the early prediction of students’ academic performance in blended learning. *Educational Technology and Society*, *21*(2), 220–232.

Luo, L., Cheng, X., Wang, S., Zhang, J., Zhu, W., Yang, J., & Liu, P. (2017). Blended learning with Moodle in medical statistics: An assessment of knowledge, attitudes and practices relating to e-learning. *BMC Medical Education*, *17*(1), 1–9. https://doi.org/10.1186/s12909-017-1009-x

Shi, Y., Tong, M., & Long, T. (2021). Investigating relationships among blended synchronous learning environments, students’ motivation, and cognitive engagement: A mixed methods study. *Computers and Education*, *168*(February), 104193. https://doi.org/10.1016/j.compedu.2021.104193

Shu, H., & Gu, X. (2018). Determining the differences between online and face-to-face student–group interactions in a blended learning course. *Internet and Higher Education*, *39*(2017), 13–21. https://doi.org/10.1016/j.iheduc.2018.05.003

Sun, Z., & Qiu, X. (2017). Developing a blended learning model in an EFL class. *International Journal of Continuing Engineering Education and Life-Long Learning*, *27*(1–2), 4–21. https://doi.org/10.1504/IJCEELL.2017.080998

Szeto, E., & Cheng, A. Y. N. (2016). Towards a framework of interactions in a blended synchronous learning environment: what effects are there on students’ social presence experience? *Interactive Learning Environments*, *24*(3), 487–503. https://doi.org/10.1080/10494820.2014.881391

Teo, T., Zhou, M., Fan, A. C. W., & Huang, F. (2019). Factors that influence university students’ intention to use Moodle: a study in Macau. *Educational Technology Research and Development*, *67*(3), 749–766. https://doi.org/10.1007/s11423-019-09650-x

Tsai, Y. Y., Chao, C. M., Lin, H. M., & Cheng, B. W. (2018). Nursing staff intentions to continuously use a blended e-learning system from an integrative perspective. *Quality and Quantity*, *52*(6), 2495–2513. https://doi.org/10.1007/s11135-017-0540-5

Wang, C. (2021). Employing blended learning to enhance learners’ English conversation: A preliminary study of teaching with Hitutor. *Education and Information Technologies*, *26*(2), 2407–2425. https://doi.org/10.1007/s10639-020-10363-5

Wang, N., Chen, J., Tai, M., & Zhang, J. (2019). Blended learning for Chinese university EFL learners: learning environment and learner perceptions. *Computer Assisted Language Learning*, *0*(0), 1–27. https://doi.org/10.1080/09588221.2019.1607881

Wang, Y., & Han, X. (2017). Institutional Roles in Blended Learning Implementation: A Case Study of Vocational Education in China. *International Journal of Technology in Teaching and Learning*, Vol. 13, pp. 16–32.

Xing, L. (2020, December). Blended Teaching Reform and Practice of Tax Law Based on TPACK Framework. In *International conference on Big Data Analytics for Cyber-Physical-Systems* (pp. 735-742). Springer, Singapore.

Yang, J., Yu, H., & Chen, N. shing. (2019). Using blended synchronous classroom approach to promote learning performance in rural area. *Computers and Education*, *141*(July), 103619. https://doi.org/10.1016/j.compedu.2019.103619

Yang, X., Zhou, X., & Hu, J. (2021). Students’ preferences for seating arrangements and their engagement in cooperative learning activities in college English blended learning classrooms in higher education. *Higher Education Research and Development*, *0*(0), 1–16. https://doi.org/10.1080/07294360.2021.1901667

Yao, C. (2017). A case study on the factors affecting Chinese adult students’ English acquisition in a blended learning environment. *International Journal of Continuing Engineering Education and Life-Long Learning*, *27*(1–2), 22–44. https://doi.org/10.1504/IJCEELL.2017.080993

Yao, C. (2019a). A case study of chinese adult learners’ english acquisition in a blended learning environment. *Australian Journal of Adult Learning*, *59*(1), 115–135.

Yao, C. (2019b). An investigation of adult learners’ viewpoints to a blended learning environment in promoting sustainable development in China. *Journal of Cleaner Production*, *220*, 134–143. https://doi.org/10.1016/j.jclepro.2019.01.290

Zhang, J. H., Zou, L. cong, Miao, J. jia, Zhang, Y. X., Hwang, G. J., & Zhu, Y. (2020). An individualized intervention approach to improving university students’ learning performance and interactive behaviors in a blended learning environment. *Interactive Learning Environments*, *28*(2), 231–245. https://doi.org/10.1080/10494820.2019.1636078

Zhang, Y., Chen, T., & Wang, C. (2020). Factors Influencing Students’ Willingness to Choose Blended Learning in Higher Education. *Lecture Notes in Computer Science (Including Subseries Lecture Notes in Artificial Intelligence and Lecture Notes in Bioinformatics)*, *12218 LNCS*, 289–302. https://doi.org/10.1007/978-3-030-51968-1_24

Zhang, Z., Cao, T., Shu, J., & Liu, H. (2020). Identifying key factors affecting college students’ adoption of the e-learning system in mandatory blended learning environments. *Interactive Learning Environments*, *0*(0), 1–14. https://doi.org/10.1080/10494820.2020.1723113

Zhou, M., & Li, Z. (2019). Blended mobile learning in theatre arts classrooms in higher education. *Innovations in Education and Teaching International*, *56*(3), 307–317. <https://doi.org/10.1080/14703297.2018.1447389>
